# Transient pseudohypoaldosteronism in infancy mainly manifested as poor appetite and vomiting: Two case reports and review of the literature

**DOI:** 10.3389/fped.2022.895647

**Published:** 2022-08-25

**Authors:** Yueerlanmu Tuoheti, Yucan Zheng, Yan Lu, Mei Li, Yu Jin

**Affiliations:** ^1^Medical School of Nanjing University, Nanjing, China; ^2^Department of Gastroenterology, Children’s Hospital of Nanjing Medical University, Nanjing, China

**Keywords:** digestive tract symptom, electrolyte disturbances, transient pseudohypoaldosteronism, urinary tract infection, urinary tract abnormalities

## Abstract

**Introduction:**

Transient Pseudohypoaldosteronism (TPHA) is a very rare condition usually secondary to urinary tract malformations (UTM) and/or urinary tract infection (UTI). It is characterized by hyperkalemia, hyponatremia, metabolic acidosis, and elevated plasma aldosterone levels. Given that the predominant manifestations of TPHA patients are digestive tract symptoms, such as poor appetite, vomiting, and weight gain, it is easily misdiagnosed as digestive tract diseases.

**Case reports:**

Two children with poor appetite and vomiting were admitted to the Department of Gastroenterology, Children’s Hospital of Nanjing Medical University, from 2020 to 2021. Laboratory test results of these two children revealed hyponatremia (< 135.00 mmol/L), hyperkalemia (> 5.50 mmol/L), and hyperaldosteronism (> 180.00 ng/L). Moreover, genetic tests demonstrated no genetic variants highly associated with the phenotype in both cases. The two patients were subsequently treated with electrolyte correction. One of them also treated with antibiotics and one of them underwent surgery. They were followed for 8 and 4 months, respectively. No complications were observed during the follow-up period. This review aimed to outline both cases with parental consent.

**Conclusion:**

Transient pseudohypoaldosteronism should be considered in children younger than 6 months, presenting with vomiting, poor appetite, unexplained hyponatremia, hyperkalemia, elevated aldosterone levels, and urethral malformation or urinary tract infection. Furthermore, attention should be paid to whether salt supplementation or anti-infection therapy is effective.

## Introduction

Transient pseudohypoaldosteronism (TPHA) is characterized by hyperkalemia, hyponatremia, and metabolic acidosis associated with high levels of plasma aldosterone and renin, chiefly secondary to urinary tract malformation (UTM) and/or urinary tract infection (UTI) (1, 2). Since the first TPHA case was described in 1983, 149 TPHA-related cases have been reported abroad, whereas only 1 case has been documented in China (1). TPHA is easily misdiagnosed as a digestive tract disease, considering most cases are characterized by digestive tract symptoms, such as nausea, vomiting, sudden cardiac arrest, acute renal damage, and adrenal crisis (1, 3). Therefore, prompt diagnosis and early treatment are vital for the prognosis of children with TPHA.

## Case reports

### Case 1

A 2-month-old full-term female baby was admitted to our hospital with a one-month history of poor feeding and weight loss. She has experienced increased irritability, frequent emesis and lost 0.5 kg in the past month. Prior to her arrival at our hospital, she was prescribed oral probiotics (Bifidobacterium bifidum 0.5 g/kg/day for 7 days). Her birth and family history were unremarkable. On admission, her weight was 4 kg (−3 SD). Her mental and nutritional status was poor. Abdominal examination revealed a palpable mass of 3 cm × 4 cm in the upper left abdomen. She was administered 10% calcium gluconate (1 ml/kg/day IV for 4 day) and furosemide (1 mg/kg/day IV for 3 days) to lower serum potassium levels, 5% sodium bicarbonate (1.5 ml/kg/day IV for 3 days) to treat metabolic acidosis, and 0.9% sodium chloride (19743.75 mEq/kg/day IV) to address hyponatremia. After 8 days of treatment, her diet and electrolyte levels improved.

At the age of 3 and 5 months, she was hospitalized twice due to recurrent poor appetite. She was given hydrocortisone sodium succinate (32.62 mg/m2/day) and 9α-fludrocortisone (0.1 mg/day). The dose of hydrocortisone sodium succinate was gradually decreased, in addition to potassium reduction and salt supplementation. However, serum aldosterone levels kept fluctuating. At the age of 6 months, her general status gradually improved, and hydrocortisone sodium succinate and 9α-fludrocortisone were discontinued. The child was diagnosed with TPHA based on the onset, clinical manifestations, and lab and imaging test results. She was followed up for 8 months after discharge. At 13 months, her body weight was 8.6 kg (−1 SD∼M), and her developmental milestones were comparable to toddlers of her age. Finally, serum sodium, potassium, and aldosterone levels were normal.

**FIGURE 1:**
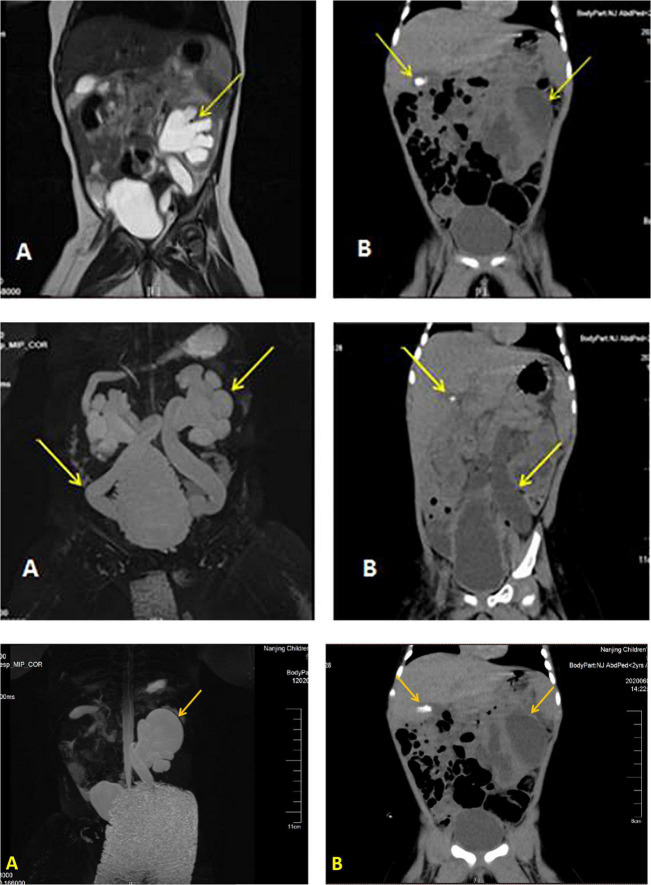
Imaging results displaying a dilated left ureter and renal pelvis and gallstone in the gallbladder. Image of the urinary MR of case 1 **(A)**, Full abdominal CT of case 1 **(B)**.

Details of the laboratory test results are presented in Table 1. Other laboratory results were consistently normal, including angiotensin II levels, cortisol, plasma rennin activity, adrenocorticotropic hormone (ACTH), cortisol, sex hormone, androstenedione, 17-hydroxyprogesterone (17-OHP), urinalysis, urine culture, and chromosome analysis. The Genetic testing results collected from the medical record system showed a heterozygous variant (*CPT2* gene c.1891 > T) was derived from the mother. However, we found no genetic variants highly associated with the phenotype. Electrocardiogram and pylorus ultrasonography findings were normal; abdominal CT scan revealed left retroperitoneal cystic foci and strong echo mass in the gallbladder in addition to abnormalities in the left kidney and ureter (Figure 1). Magnetic resonance urography (MRU) illustrated posterior renal cystic lesions associated with left calyces, left hydronephrosis, and left tortuous dilation of the ureter (with distal ureteral obstruction).

**TABLE 1:** Timeline with relevant data of the two transient pseudohypoaldosteronism cases.

Relevant data	Case 1				Case 2		Normal range
	2 months	3 months	5 months	13 months	7 months	11 months	
Cardinal symptom	Poor feeding and weight loss	Poor appetite	Poor appetite and vomiting	None	Vomiting and fever	None	-
Serum potassium (mmol/L)	6.54	6.15	6.01	4.6	8.34	4.30	3.50∼5.50
Serum sodium (mmol/L)	114.30	129.90	126.60	135.0	114.00	142.0	135.00∼145.00
Serum chlorine (mmol/L)	86.80	97.80	94.50	112.0	79.00	107.6	96.00∼108.00
Serum aldosterone (ng/L)	3.63	3460.18	4216.83	146.92	1036.50	240.01	General food: 30.00∼180.00
Renin activity (ng/ml/h)	1.02	0.54	0.56	0.44	3.35	0.57	General food: 0.13∼1.74
PH	7.37	7.34	7.32	7.32	7.32	7.30	7.35∼7.45
HCO_3_^–^ (mmol/L)	18.80	19.60	18.40	17.9	15.80	19.2	22.00∼28.00
Treatment	10% calcium gluconate, furosemide, 5%sodium bicarbonate, 0.9% sodium chloride	Potassium reduction, salt supplementation, hydrocortisone sodium, succinate oral saline, 9α-fludrocortisone	α-fludrocortisone, hydrocortisone acetate	None	10% calcium gluconate, 5% sodium bicarbonate, insulin	None	

### Case 2

A 7-month-old full-term male baby was admitted to our hospital with a 6–day history of vomiting and fever. The child received oral amoxicillin (150 mg/kg/day) in another hospital, but his condition did not improve. He had a history of bronchitis and recurrent eczema in the past 7 months, and his birth and family history were unremarkable. On admission, his body weight was 8 kg (−1SD∼M). His condition improved with the administration of 10% calcium gluconate (0.125 ml/kg/day IV single-dose), 5% sodium bicarbonate (0.75 ml/kg/day IV for 3 days), insulin (0.19 IU IH single-dose), sodium chloride supplementation (7678.12 mEq/kg/day PO and 9871.87 mEq/kg/day IV for 4 days), and intravenous meropenem (0.16 g/kg for 16 days). After 20 hours of treatment, serum potassium levels returned to normal, whereas hyponatremia persisted for 4 days. Following discharge, his plasma aldosterone level was marginally above average (240.01 ng/L). On the 19th day of admission, the patient underwent UTM correction (cystoscopy posterior urethral valvulotomy). To prevent reinfection, nitrofurantoin (1.56 mg/kg/day) was taken orally for 2 weeks, and the patient was followed up for 4 months. At 11 months, his weight was 11 kg, with normal motor development typical for a healthy child; indeed, he did not exhibit signs of TPHA during the 4-month follow-up.

**FIGURE 2:**
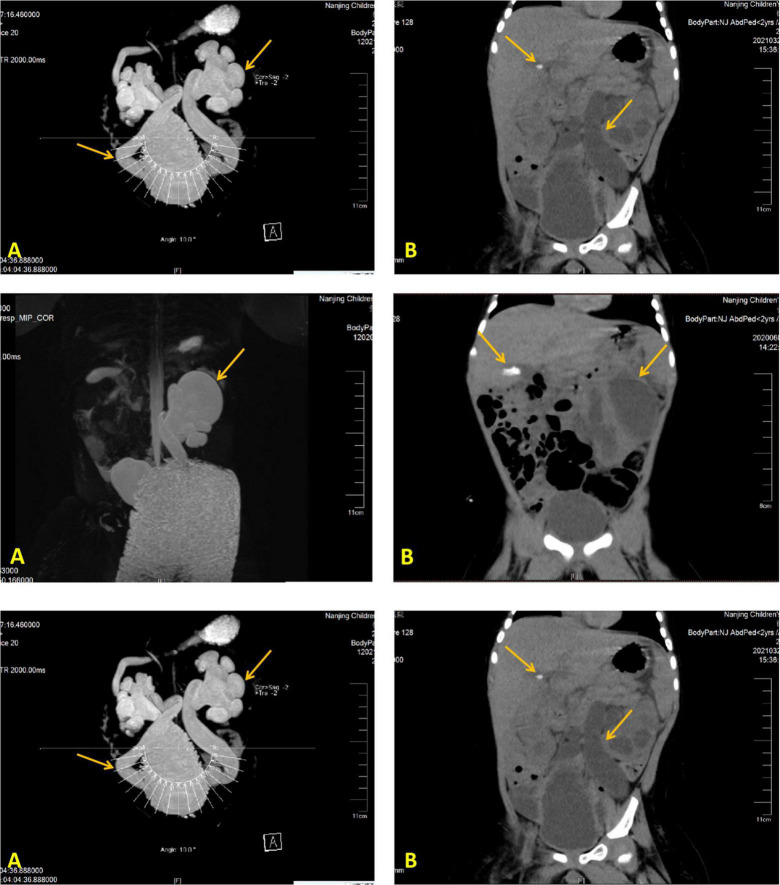
Imaging modalities and results illustrating a dilated left ureter and renal pelvis and gallstone in the gallbladder. Image of the urinary MR of case 2 **(A)**, Full abdominal CT of case 2 **(B)**.

His laboratory test results during hospitalizations are summarized in Table 1; the remaining aberrant laboratory results were as follows: angiotensin II 145.88 ng/L, cortisol 756.90 nmol/L, urine microalbumin 33.70 mg/L, urine leukocyte count 1321.30/μL, urine bacteria 2103.30/μL, Morganella morganella subspecies cultured in urine, CRP 98 mg/L, WBC 28.75 × 10^9^/L, procalcitonin (PCT) 1.15 ng/mL, and HGB 118 g/L. Besides, expected laboratory results were sex hormones, ACTH levels, and kidney function indexes. The genetic test results of case 2 showed that he got heterozygote of these variants: c.1968 + 41G > A in the *ABCB6* gene, c.1234C > G in the *ARMC5* gene, c.4898T > A in the *COL4A3* gene, c.1809G > C in the *GPD2* gene, c.1543C > T in the *HNF1B* gene, c.2392C > T in the *MLL5* gene, c.1279 G > A in the *TFAP2A* gene, c.8764G > A in the *TNXB* gene, and c.1978A > G in the *IGSF1* gene. We found no intentional variants in the genes related to primary PHA of the child, including *ABCB6 SCNN1A, SCNN1B, SCNN1G, NR3C2, WNK1, WNK4*, and *CUL3* gene. Abdominal CT scan results displayed bilateral hydronephrosis with obstruction of the ureter close to the bladder and gallstone (Figure 2); urinary tract (MRI and MRU) imaging showed bilateral hydronephrosis with a dilated ureter, as well as vesicoureteral reflux.

## Discussion

Pseudohypoaldosteronism (PHA) is a group of diseases characterized by electrolyte disorders, metabolic acidosis, and an elevated serum aldosterone level (1–3). It can be divided into primary pseudohypoaldosteronism and secondary pseudohypoaldosteronism (SPHA) according to etiology (2). The latter, also referred to as transient pseudohypoaldosteronism (TPHA), is a condition in which the renal tubule is unresponsiveness to aldosterone (1–3). Therefore, TPHA clinically manifests as transient and severe hyponatremia, hyperkalemia, metabolic acidosis, an elevated serum aldosterone level, and an elevated or normal renin activity (1–9).

It was previously established that TPHA in infants could be caused by multiple factors, including urinary tract malformations, urinary tract infection, and drugs (2, 10–14). Delforge et al. (3) described that TPHA caused by urethral malformation with urinary tract infection is more prevalent, but TPHA caused by unilateral UTM without UTI was rarely encountered. In case 2, bilateral ureteral obstruction was present, bacteria were cultured in the urine sample, and increased CRP levels and PCT and WBC counts implied the presence of a urinary tract infection. Therefore, case 2 was identified as TPHA resulting from bilateral UTM and UTI. Case 1 was considered a case of TPHA induced by unilateral urinary tract malformations (UTM) since results of imaging tests displayed distal obstruction of the left ureter with pyelogenic cysts, and there was no evidence of urinary tract infection.

The pathogenesis of TPHA remains ambiguous, but three factors, including age, urinary tract obstruction, and urinary tract infection, may be the keys to the development of TPHA (1, 3, 11). (1) Studies have validated that TPHA is more common in infants younger than 6 months, which may be related to their immature renal tubules (3, 15–17). (2) In children with UTM, urinary tract obstruction or vesicoureteral reflux leads to increased intrarenal pressure, which results in the down-regulation of aldosterone receptors (2, 5, 14). These two factors also lead to a surge in the intrarenal synthesis of various cytokines, such as tumor necrosis factor-β1 (TGF-β1), tumor necrosis factor-α (TNF-α), etc. The former can inhibit the action of aldosterone (3, 5, 15, 18, 19). (3) In children with UTI, internal and external toxins not only directly damage aldosterone receptors but also stimulate the immune system to generate inflammatory factors, such as interleukin-1 (IL-1), thromboxane, natriuretic peptide, etc. (15, 16, 18). TPHA has various clinical manifestations, most prominently manifesting as digestive symptoms, including poor feeding, vomiting, abdominal distension, and diarrhea (1, 13, 15). Children with TPHA may develop adrenal crisis, acute renal failure, and cardiac arrest associated with severe electrolyte disturbances (1, 12, 16, 18). Furthermore, researchers have reported cases of TPHA combined with pneumothorax and cholecystolithiasis. The pathogenesis of pneumothorax is still elusive, whereas cholecystolithiasis may be linked to chronic dehydration in children (1, 16). Herein, CT of the whole abdomen suggested that the two cases were complicated with gallstone.

Clinically, the symptoms of children with TPHA are comparable to sepsis or pyloric stenosis, but the differential diagnosis is straightforward, and the workup includes blood culture and pyloric ultrasound (5, 13). Nonetheless, several diseases, such as primary PHA, congenital adrenal hyperplasia (CAH), and congenital adrenal hypoplasia, typically present as severe and persistent hyponatremia, hyperkalemia, and metabolic acidosis and are clinically challenging to differentiate from TPHA (2, 8, 18, 20). Chromosome analysis and relevant genetic testing can be employed to identify this disease.

Transient pseudohypoaldosteronism treatment focuses on correcting electrolyte imbalance and acidosis, followed by the administration of antibiotics, correcting malformations, and addressing complications (3, 19). In children with a salt loss crisis, 9α-fluorohydrocortisone and hydrocortisone can be given prior to the diagnosis being made (2, 21). The administration of sodium, potassium, and UTM/TUI correction is effective in the majority of infants. Consequently, it is necessary to regularly monitor aldosterone levels, serum electrolytes, blood pH, and body weight and follow up for at least 1 year (2, 12, 19).

## Conclusion

In summary, this study reported two cases of TPHA with vomiting and loss of appetite as the chief manifestations. TPHA should be considered in children under 6 months of age presenting with symptoms of vomiting, poor appetite, and weight loss. Finally, serum electrolyte levels, urinary tract ultrasound, and genetic testing should be performed on these patients.

## Data availability statement

The original contributions presented in this study are included in the article/supplementary material, further inquiries can be directed to the corresponding authors.

## Ethics statement

The studies involving human participants were reviewed and approved by the Ethics Committee of Children’s Hospital of Nanjing Medical University. Written informed consent to participate in this study was provided by the participants’ legal guardian/next of kin. Written informed consent was obtained from the individual(s), and minor(s)’ legal guardian/next of kin, for the publication of any potentially identifiable images or data included in this article.

## Author contributions

YT and YZ contributed to the study design, data collection, analysis, and manuscript preparation. YL contributed to data analysis, manuscript preparation, and revised the manuscript. ML and YJ contributed to the study design and critically revised the manuscript. All authors contributed to the article and approved the submitted version.
